# Fibrillary Glomerulopathy with a High Level of Myeloperoxidase-ANCA: A Case Report

**DOI:** 10.1155/2020/6343521

**Published:** 2020-03-23

**Authors:** Tomohiko Asakawa, Mea Asou, Shigeo Hara, Takashi Ehara, Makoto Araki

**Affiliations:** ^1^Department of Internal Medicine, Suwa Central Hospital, Chino, Nagano 391-8503, Japan; ^2^Department of Diagnostic Pathology, Kobe City Medical Center General Hospital, 2-1-1, Minatojima Minamimachi, Chuo-ku, Kobe 650-0047, Japan; ^3^Graduate School of Health Sciences, Matsumoto University, 2095-1 Niimura, Matsumoto 390-1241, Japan

## Abstract

An elderly woman was admitted with the chief complaint of gross hematuria. Laboratory values indicated a high myeloperoxidase-ANCA level. In renal histological examination, 40% of the glomeruli showed crescent formation, but immunofluorescence staining showed positivity for IgG, C3, and C1q. Furthermore, the deposition of fibrils in the glomerulus was noted on electron microscopy, and immunohistochemical staining showed strong positivity for DNA-J heat shock protein family member B9 (DNAJB9). Crescent formation is a common feature of fibrillary glomerulonephritis (FGN). Thus, in ANCA-positive crescentic glomerulonephritis, immunohistochemical assessments for immunoglobulins and DNAJB9, as well as electron microscopy, are important to correctly diagnose FGN.

## 1. Introduction

Gross hematuria with renal dysfunction suggests the possibility of rapidly progressive glomerulonephritis (RPGN) and requires urgent attention. Anti-neutrophil cytoplasmic antibody- (ANCA-) associated vasculitis (AAV) represents RPGN and is characterized by elevated serum ANCA levels and pauci-immune crescentic glomerulonephritis. But it is well known that the presence of ANCA is not highly specific for the diagnosis of vasculitis.

Fibrillary glomerulonephritis (FGN) is a rare glomerular disease characterized by noncongophilic amyloid-like fibrillary glomerular deposits. The histological type of FGN is the mesangial proliferating glomerulonephritis pattern, but it is often accompanied by crescents. FGN has a poor prognosis and often results in relatively rapid deterioration of renal function, like RPGN. The diagnosis could be made only by electron microscopy. But recently a simple immunostaining method has been established. Thus, the number of cases may increase in the future.

RPGN due to AAV is known as pauci-immune crescent glomerulonephritis, but in fact, immunoglobulin deposition is observed in many cases [1–3]. There may be cases of FGN with crescents among them. But, if electron microscopy is not performed in such cases, differentiation between the two is impossible.

## 2. Case Presentation

**Figure 1:**
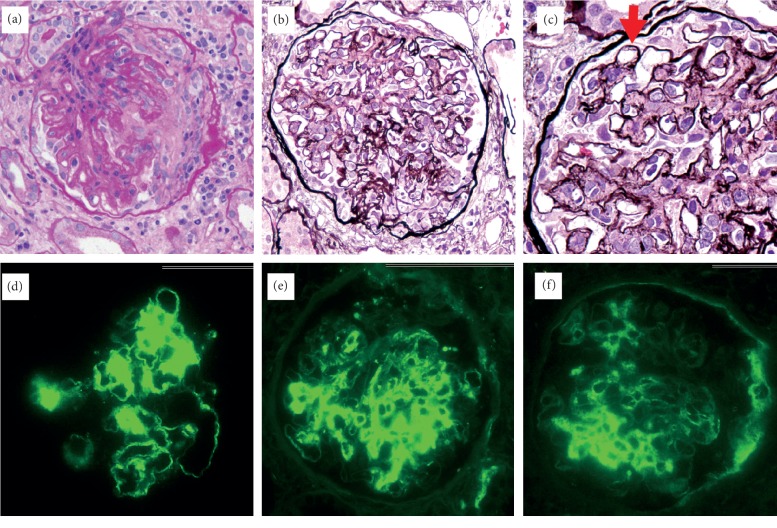
Light microscopic appearance. Periodic acid–Schiff (PAS) staining showed crescents formation (a) (×200). Periodic acid methenamine silver (PAM) staining showed mesangium expansion (b) (×200) and segmental duplication of glomerular basement membrane (c) (×200 arrow). Positive immunofluorescent staining of IgG (d), C3 (e), and C1q (f). IgM and IgA were negative.

**Figure 2:**
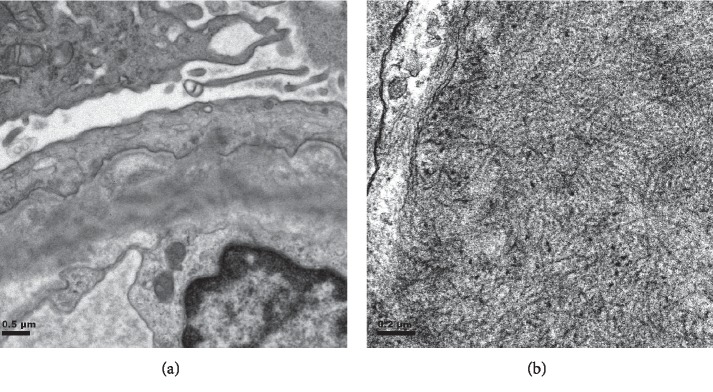
Electron micrographs of a glomerular tuft. (a) Electron microscopic showed the presence of electron-dense deposits in the glomerular mesangium and capillary walls. (b) Higher magnification reveled deposits as random arrangement of nonbranching fibrils (×7,000 and ×20,000).

**Figure 3:**
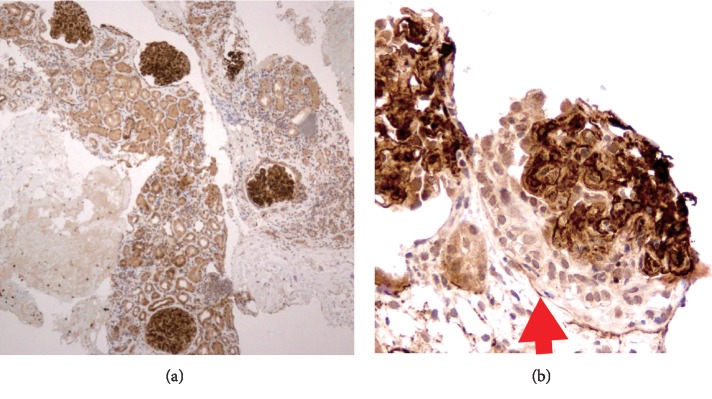
Immunohistochemistry of DNAJB9. (a) Glomeruli are strongly stained (×40). (b) In contrast to the glomerulus, crescent (arrow) is not stained (×400).

A 71-year-old woman was admitted with the chief complaint of gross hematuria. Her laboratory values indicated a creatinine (Cr) level of 1.72 mg/dl and massive proteinuria of 5.1 g/gCr. There was no fever, and her white blood cells were within the normal range. Further examination revealed a high myeloperoxidase (MPO)-ANCA level (125 U/ml, normal < 3.5), no monoclonal proteins, and normal complement protein levels (Table 1). Thus, a renal biopsy was performed; it showed mesangial proliferation in all 13 glomeruli and crescent formation in 5 glomeruli. Immunofluorescence staining showed positivity for immunoglobulin G (IgG), C3, and C1q (Figure 1). The deposition of fibrils was noted in the glomerulus on electron microscopy (Figure 2). Additional staining showed negativity for Congo red, and there was no kappa/lambda (*κ*/*λ*) imbalance. In addition, immunohistochemical staining for DNA-J heat shock protein family member B9 (DNAJB9, Anti-DNAJB9 primary antibody was purchased from Proteintech (rabbit polyclonal antibody; catalog no. 13157-1-AP)) showed strong positivity in the glomerulus (Figure 3). These results indicated FGN.

The optimal treatment modalities were unclear based on the results primarily owing to the fact that it still remained to be determined whether the patient had FGN alone or FGN accompanied by AAV. Since there is no treatment specifically recommended for FGN, induction therapy for AAV was initiated with high-dose oral prednisone (PSL) 1 mg/kg/day and intravenous cyclophosphamide pulse therapy (IVCY, 7.5 mg/kg/day, biweekly). After 1 month, IVCY was switched to azathioprine (AZA) as renal function improved with a Cr level of 1.05 mg/dl and urinary protein of 3.0 g/gCr, and inflammatory response was negative. Two months later, she developed manic depression. Thus, AZA was discontinued and PSL was reduced to 5 mg/day because of suspected side effects of the drugs.

One and a half years later, renal function had not deteriorated; the Cr level was 0.84 mg/dl and urinary protein was 0.6 g/gCr. The titer of ANCA also remained at a low level of 10.7 IU/ml, and the patient's mental condition was stable with the administration of valproic acid.

## 3. Discussion

We encountered a case of elevated serum MPO-ANCA level and anti-DNAJB9 positive staining indicating glomerulonephritis with crescent formation. FGN is a rare glomerular disease noted in 0.6–1% of all kidney biopsies. The clinical features of FGN, usually present in older adults and include proteinuria, hematuria, and hypertension [4–6], which are similar to those of AAV. In renal biopsy, AAV is characterized by crescent formation, but about 25% of cases of FGN also show crescents. The hallmark of renal damage caused by AAV is pauci-immune, which is very difficult to determine by immunostaining when FGN is in the background. To our knowledge, FGN with serum MPO-ANCA positivity, like in our case, is very rare.

Although the etiology of FGN is still unknown, diagnostic criteria based on morphological features have been proposed. This case fulfilled the following previously-established diagnostic criteria [7, 8]: glomerular deposition of fibrils that were (i) randomly oriented, (ii) lacked hollow centers at magnification of <30,000, (iii) were Congo-red negative, and (iv) stained positively with antisera to immunoglobulins on immunofluorescence staining. DNAJB9 was identified as a biomarker of FGN in 2017 [6–9]. DNAJB9 is found in the endoplasmic reticulum (ER) and functions as a co-chaperone for heat shock protein 70 (Hsp 70). Its role appears to be ensuring proper folding of proteins. DNAJB9 is ubiquitously expressed. Immunohistochemically, DNAJB9 shows weak granular expression in the cytoplasm of tubular epithelial cells, glomerular cells, and vascular smooth muscle cells in normal renal tissues. In FGN, there is prominent staining for DNAJB9 outside the cell of the glomerulus, making it easy to distinguish from its expression pattern in normal tissues. In fact, in this case, there were apparent staining differences between the renal tubules and the mesangial region. DNAJB9 immunostaining is known to be positive in 98–100% of FGN cases [6, 8] and is expected to be a new diagnostic tool to replace electron microscopy in such cases.

FGN can present morphologically with varying patterns of glomerular injury, including crescent formation. In FGN, the mesangial proliferative glomerulonephritis pattern is seen in as many as 70% of the cases [10], and the endocapillary proliferative glomerulonephritis, segmental membranous nephropathy, and diffuse sclerosing glomerulonephritis patterns are also recognized. Crescent formation is common and has been observed in 17–31% of patients [4, 7, 11]. Glomerular damage due to FGN including crescent formation is thought to be caused by an immune complex-type renal disorder. FGN shows immunofluorescence staining for deposition of C3 (92%), C1q (36%), as well as IgG (100%, significant for IgG1 and IgG4) [8]. Although FGN may be classified as one of the monoclonal gammopathies of renal significance (MGRS), only approximately 11% of cases show *κ* or *λ* light-chain restriction [4]; that is, most FGN cases are characterized by polyclonal IgG deposits, as this case. Furthermore, only 2% of the patients have hypocomplementemia. Despite fibrils and IgG coexist, whether fibrils are composed of IgG remains unclear, and there is a possibility that the fibril deposition is secondary to an immune response [12].

A small number (2/34) of p-ANCA-positive FGN cases with crescents have been reported [7]. However, because diseases other than AAV are sometimes positive for ANCA [13], the interpretation of its staining is difficult except in typical vasculitis. In addition, systemic diseases such as autoimmune diseases, malignant tumors, and chronic infections are known to be associated with 1/3^rd^ of FGN cases [14]. The pathogenicity of ANCA is thought to be due to differences in its epitopes [15]; however, there is no ideal way to distinguish between them. Furthermore, AAV with immune complex deposition is often experienced [1–3]. If ANCA is elevated serologically and crescents are present, FGN may be missed because electron microscopic examination is infrequent. Since FGN can be diagnosed by immunohistochemical staining with the advent of DNAJB9, it is possible to increase the number of reports that the ANCA-associated glomerulonephritis associated with immune complex deposition was actually FGN. The etiology of FGN is not yet known, but it is hypothesized to be an ER stress/misfolding-related disease. Although immune responses and inflammation are known to be associated with ER stress, the relationship between ANCA and FGN remains unclear [16].

FGN is difficult to treat, and half of the patients progress to end-stage renal disease within 4 years of diagnosis [4, 5, 17, 18]. However, some cases of spontaneous cure have been reported [19]. The standard treatment of FGN has not been established; nonspecific immunosuppressive therapy targeting immune-complexes is being attempted, and there is much overlap between treatment modalities for AAV and FGN. Specific therapies for FGN are expected to be developed in the future based on its association with DNAJB9.

## 4. Conclusion

Based on the histological findings, it was considered that the patient's renal dysfunction was caused by either FGN alone or from concomitant AAV and FGN. There is no ideal way to know if ANCA has pathological significance, and no conclusion could be reached based on this case. Altogether, in cases of ANCA-positive crescentic glomerulonephritis, if immunostaining reveals immunoglobulin deposition, the diagnosis of FGN may be missed if electron microscopy or DNAJB9 staining is not performed.

## Figures and Tables

**Table 1:** Laboratory findings on admission.

		Unit
WBC	3910	/*μ*L
Hb	7.7	g/dL
Plt	32.2	10^4^/*μ*L
TP	6	g/dL
Alb	2.6	g/dL
GOT	17	IU/L
GPT	8	IU/L
LDH	224	IU/L
T-Bil	0.35	mg/dL
ALP	174	IU/L
r-GTP	11	IU/L
HbA1C	5.8	％
BUN	23.3	mg/dL
Cr	1.72	mg/dL
Na	139.3	mEq/L
K	3.6	mEq/L
Cl	106	mEq/L
CRP	0.59	mg/dL
HBs-Ag		(−)
HCV-Ab		(−)
C3	115	mg/dL
C4	33	mg/dL
CH50	30.5	U/ml
ANA	×40	
PR3-ANCA	<1.0	U/mL
MPO-ANCA	125	IU/mL
Serum immunofixation electrophoresis	M protein (−)	
Urine immunofixation electrophoresis	M protein (−)	
FLC (*κ*/*λ* ratio)	0.97	
Uric protein	(3+)	
Uric protein	5.1	g/gCr
Uric blood	(3+)	
Urine red blood cells	55∼99	/HPF
Oval fat body	(+)	
Granular cast	1∼10	/WF
Waxy cast	1∼10	/WF

FLC; free light chain, HPF; high power field, WF; whole field.
